# 单倍体造血干细胞移植后Wernicke脑病3例报告并文献复习

**DOI:** 10.3760/cma.j.cn121090-20231214-00309

**Published:** 2024-08

**Authors:** 倩倩 肖, 晓林 于, 晓晨 宋, 文君 李, 磊 邓, 怡西 侯, 芳 周

**Affiliations:** 1 山东第二医科大学临床医学院，潍坊 261053 School of Clinical Medicine, Shandong Second Medical University, Weifang 261053, China; 2 解放军联勤保障部队第九六〇医院血液病科，济南 250031 Department of Hematology, The 960th Hospital of The People's Liberation Army (PLA) Joint Logistics Support Force, Jinan 250031, China

## Abstract

例1，女，27岁，ALK阳性间变性大细胞淋巴瘤/白血病（ALCL）；例2，男，27岁，急性髓系白血病；例3，男，56岁，骨髓增生异常综合征。3例患者单倍体造血干细胞移植后长期伴有重度口腔黏膜炎、恶心、呕吐、腹泻等症状，导致进食明显受限，分别于移植后50、38、50 d出现神经、精神症状，经头颅磁共振成像检查诊断为Wernicke脑病，经维生素B_1_静脉滴注治疗后病情明显好转。

## Abstract (en)

Case 1: A 27-year-old female with ALK-positive anaplastic large cell lymphoma/leukemia; Case 2: A 27-year-old male with acute myeloid leukemia; Case 3: A 56-year-old male with myelodysplastic syndrome. These three patients underwent haploid hematopoietic stem cell transplantation and experienced severe oral mucosal inflammation, nausea, vomiting, diarrhea, and other symptoms over a long period, which significantly restricted eating. Neurological and psychiatric symptoms appeared at 50, 38, and 50 days following transplantation, respectively. The diagnosis of Wernicke encephalopathy was made by head magnetic resonance imaging, whereas the condition improved significantly after intravenous infusion of vitamin B_1_.

Wernicke脑病（Wernicke encephalopathy, WE）是一种维生素B_1_（硫胺素）缺乏引起、以“眼肌麻痹、意识障碍和共济失调”三联征为主要表现的中枢神经系统（CNS）代谢性脑病，多见于长期酗酒者，也可见于胃肠道疾病、妊娠剧吐、长期肠外营养等人群[1]。造血干细胞移植（HSCT）患者由于大剂量放化疗预处理及移植后胃肠道移植物抗宿主病（GVHD）等因素，往往引起厌食、恶心、呕吐、腹泻及口腔黏膜炎等并发症，存在维生素B_1_缺乏的风险。随着HSCT技术的迅速发展，移植后CNS并发症越来越被关注，但主要集中于治疗相关不良反应、感染、免疫反应、脑血管疾病及原发疾病浸润等[2]。本文报告3例单倍体造血干细胞移植（haplo-HSCT）后合并Wernicke脑病患者的诊疗过程并进行相关文献复习，旨在提高血液科医师对于HSCT后Wernicke脑病的认识。

## 病例资料

例1，女，27岁，2022年4月诊断为“ALK阳性间变性大细胞淋巴瘤/白血病（ALCL）”，行8个疗程化疗并以多种新药治疗后获得完全缓解（CR），但15 d后复发，外周血淋巴瘤细胞占1.57％。于2023年4月行haplo-HSCT，预处理采用TBI/Cy（全身照射治疗/环磷酰胺）+阿糖胞苷+米托蒽醌脂质体+司莫司汀方案，期间出现重度口腔黏膜炎、腹腔感染，以广谱抗生素治疗后好转。单个核细胞（MNC）输注量为32.39×10^8^/kg，CD34^+^细胞输注量为8.27×10^6^/kg。+9 d出现上消化道出血，予禁食、抑酸、止血等治疗。+15 d粒系造血重建。+18 d复查骨髓象为CR，微小残留病（MRD）阴性，短串联重复序列（STR）检测示完全嵌合。+19 d起反复恶心、呕吐，腹胀、纳差明显，考虑Ⅱ度急性移植物抗宿主病（aGVHD），加用甲泼尼龙抗GVHD治疗，同时肠外营养支持治疗，患者逐渐出现极度消瘦等恶病质表现。+50 d诉头晕、视物模糊，双眼发黑，颅脑CT未见明显异常，腰穿脑脊液未见异常，+52 d出现眼球震颤、烦躁、意识淡漠、记忆力减退等症状，头颅磁共振成像（MRI）示：中脑导水管周围及双侧丘脑见对称性斑片状长T1长T2信号，T2加权像液体衰减反转恢复（T2-FLAIR）呈高信号，弥散加权成像（DWI）呈略高信号，考虑Wernicke脑病（图1）。给予维生素B_1_ 200 mg每日3次静脉滴注治疗。+56 d，头晕、视物模糊、眼球震颤明显好转。+64 d，烦躁、意识淡漠等精神症状基本消失，记忆力逐渐恢复，头颅MRI示异常信号减低、范围缩小。+119 d头颅MRI示异常信号基本消失。随访至移植后9个月，患者无神经、精神症状，记忆力基本恢复，眼球震颤基本消失。

**图1:**
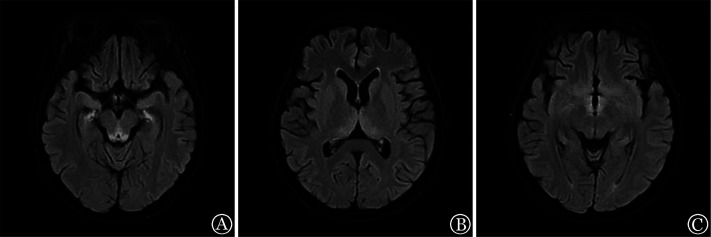
Wernicke脑病患者（例1）头颅磁共振成像表现 **注** 中脑导水管周围（A）、双侧丘脑（B）、第三脑室周围（C）T2-FLAIR对称性高信号

例2，男，27岁，2022年12月诊断为急性髓系白血病，予2个疗程IA方案（伊达比星+阿糖胞苷）、1个疗程中剂量阿糖胞苷方案化疗，获得形态学CR，MRD阴性，于2023年4月行haplo-HSCT。预处理采用Bu/Cy（白消安/环磷酰胺）+阿糖胞苷+地西他滨方案，期间出现发热、腹泻、腹痛等症状，以广谱抗生素抗感染治疗后好转。MNC输注量为29.64×10^8^/kg，CD34^+^细胞输注量为3.85×10^6^/kg。移植后合并重度口腔黏膜炎，进食明显受限。+15 d粒系造血重建。+21 d起反复恶心、呕吐，考虑Ⅱ度aGVHD，加用甲泼尼龙抗GVHD治疗，效果差。+25 d复查骨髓象为CR，MRD阴性，STR示完全嵌合。+38 d诉头痛、耳鸣，头颅CT未见异常，耳鼻喉科会诊考虑感音神经性耳聋。+39 d出现反应迟钝、言语欠流利、大小便失禁，+42 d出现嗜睡，+46 d出现浅昏迷、四肢震颤及双眼球明显震颤，头颅MRI示内侧丘脑、第三脑室及中脑导水管周边可见对称性T2-FLAIR及DWI序列高信号，考虑Wernicke脑病（图2）。给予维生素B_1_ 200 mg每日3次静脉滴注，3 d后神志恢复，可简单对答，大小便正常，仍有记忆力减退。+58 d，头颅MRI示异常信号大部分消失。+68 d言语流畅，记忆力恢复，仍有双眼球震颤，头颅MRI示异常信号范围继续缩小。+90 d记忆力基本恢复，双眼球轻微震颤，头颅MRI未见异常信号。随访至移植后9个月余，仍有双眼眼球轻微震颤。

**图2:**
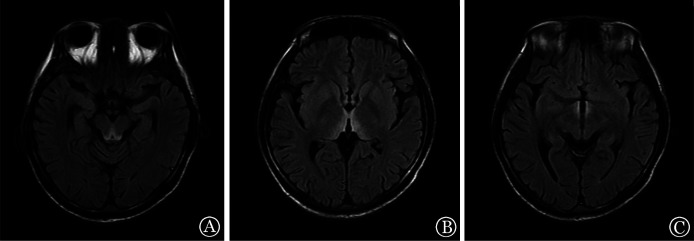
Wernicke脑病患者（例2）头颅磁共振成像表现 **注** 中脑导水管周围（A）、双侧丘脑（B）、第三脑室周围（C）T2-FLAIR对称性高信号

例3，男，56岁，2023年8月确诊骨髓增生异常综合征，2023年9月行haplo-HSCT。预处理采用Bu/Cy+阿糖胞苷+地西他滨方案。MNC输注量为23.85×10^8^/kg，CD34^+^细胞输注量为4.51×10^6^/kg。+22 d粒系造血重建，MRD 0.06％，STR检测示混合嵌合状态（T分选92.55％）。+27 d再次回输供者外周血干细胞（MNC 8.17×10^8^/kg，CD34^+^细胞数1.13×10^6^/kg）。+28 d口腔溃疡、进食受限。+30 d出现腹泻，每日3～4次，口腔溃疡继续加重，考虑Ⅱ度aGVHD，加用甲泼尼龙抗GVHD治疗，效果差，予芦可替尼抗GVHD治疗。+50 d出现视物模糊。+56 d MRD阴性，STR检测示完全嵌合，同日患者视物模糊加重伴眼球内聚、双眼球震颤、双眼外转功能受限，眼科会诊考虑眼肌麻痹。头颅CT示脑内小缺血灶，头颅MRI示多发腔隙性脑梗死、缺血灶，不考虑急性脑血管病。+58 d腰穿脑脊液未见异常。考虑不除外GVHD，给予甲泼尼龙抗GVHD治疗，效果差。+61 d患者出现反应迟钝、认知障碍，继之嗜睡、浅昏迷。头颅MRI示双侧丘脑、第三脑室、中脑导水管周围、双侧乳头体及延髓后缘见对称性斑片状长T1长T2信号，T2-FLAIR序列示高信号，DWI呈略高信号，考虑Wernicke脑病（图3）。给予维生素B_1_ 200 mg每日3次静脉滴注治疗。同日下午认知障碍好转、眼球震颤减轻，可简单语言交流，但记忆力仍有减退。+69 d患者障碍进一步好转，可正确回答问题，记忆力减退有所改善，仍有轻微眼球震颤，头颅MRI示异常信号明显缩小。随访至移植后4个月，头颅MRI示异常信号消失，神经、精神症状基本消失，仍有轻度记忆力减退。

**图3:**
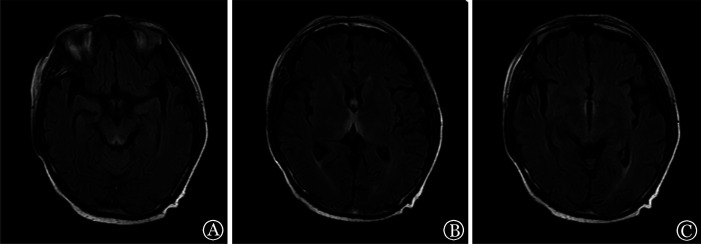
Wernicke脑病患者（例3）头颅磁共振成像表现 **注** 中脑导水管周围及双侧乳头体（A）、双侧丘脑（B）、第三脑室周围（C）T2-FLAIR对称性高信号

## 讨论及文献复习

Wernicke脑病是维生素B_1_缺乏引起的中枢神经系统代谢性脑病，1881年由波兰神经病学家Carl Wernicke发现并命名，以眼肌麻痹、意识障碍和共济失调为主要临床表现，但通常仅有约16％的患者表现为典型的三联征，多数患者仅出现1～2个症状[3]。国外尸检发现Wernicke脑病的患病率为0.8％～2.8％，但临床诊断率仅为0.04％～0.13％[4]。

维生素B_1_是三羧酸循环的重要辅酶，主要参与糖的有氧酵解。人体不能合成维生素B_1_，只能通过外界摄取，其吸收的主要部位是十二指肠。造血干细胞移植的患者，预处理过程中接受大剂量放化疗，极易出现恶心、呕吐、厌食等消化道症状，移植后GVHD、感染等并发症进一步加重胃肠道反应，抗GVHD长期使用糖皮质激素和免疫抑制剂，以及低蛋白血症、使用利尿剂等，都增加了维生素B_1_缺乏的风险[5]。

移植后Wernicke脑病的诊断主要依赖于长期营养缺乏的病史、典型临床表现及头颅MRI表现。根据欧洲神经学会联合会（ENFS）指南，Wernicke脑病的诊断需符合以下4条中的2条：饮食缺乏，眼部症状，小脑功能障碍，精神状态改变或轻度记忆力障碍[1]。Wernicke脑病主要累及丘脑内侧、第三脑室、第四脑室、中脑导水管周围灰质及乳头体等葡萄糖氧化代谢率高的部位，从而导致三联征表现[6]。仅少数患者表现为典型的三联征，临床表现往往多样化。其中眼部症状主要表现为眼球震颤、视力下降、复视等；精神状态异常主要表现意识淡漠、反应迟钝、定向力和记忆力障碍、嗜睡、昏迷等；共济失调主要表现为站立行走不稳、步态异常等。还有一些患者表现为听力下降、低体温、低血压、心动过速、癫痫发作等非特异性症状。还有学者提出，HSCT后Wernicke脑病的患者，“杨梅舌”往往早于精神障碍出现，可作为早期诊断的依据[7]。我们报告的3例患者都有眼部症状、意识障碍，而无共济失调，也未观察到“杨梅舌”表现。而且3例患者起初都只表现为头晕、耳鸣、视物模糊等不典型症状，迅速出现眼球震颤、反应迟钝、意识障碍等症状才引起重视。头颅MRI检查是Wernicke脑病最重要、最有效的工具，且MRI显示的损害范围可以反映疾病严重程度[8]。Wernicke脑病急性期，MRI主要表现为丘脑、第三脑室、第四脑室基底部、中脑导水管周围及乳头体对称性异常信号，即T1加权像低信号，T2-FLAIR和DWI高信号。T1加权像增强后乳头体强化是特征性表现[4]。研究表明，头颅MRI对于诊断Wernicke脑病特异性为93％，灵敏度为53％[9]。我们报告的第3例患者，开始出现视物模糊、眼球震颤的症状时，MRI并未见Wernicke脑病的特征性表现，至出现意识障碍时复查MRI才出现特征性改变。因此，头颅MRI检查出现上述特征性表现时，基本可以明确诊断；但头颅MRI正常时，也不能排除Wernicke脑病。此时，长期营养缺乏的病史及临床表现的早期识别格外重要。头颅CT对Wernicke脑病的诊断价值不大，检出率仅为13％[4]。本研究3例患者的头颅CT检查均未见明显异常，最终通过头颅MRI检查明确诊断。

一项对于HSCT后合并CNS并发症的1 000例患者的回顾性研究中，Wernicke脑病的发生率为1％[10]。另一项对180例骨髓移植患者的回顾性尸检分析显示，Wernicke脑病的发生率为5.5％[11]。因此，HSCT后Wernicke脑病并不罕见，但诊断率却很低，国内外相关报道及研究少见[2],[12]–[14]。其原因包括多数患者临床表现并不典型，血液科医师对于该病的认识和重视程度不够。另外，Wernicke脑病常出现在移植后早期，此时极易合并颅内感染、急性脑血管病、GVHD等CNS并发症，有时往往难以鉴别，从而导致误诊及漏诊。

Wernicke脑病对维生素B_1_反应迅速，发病初期给予维生素B_1_治疗，1周内即可观察到症状明显改善，但治疗时间通常需要1～3个月，而且常可遗留眼球震颤、共济失调等神经系统功能障碍[8]，仅有约20％的患者完全康复[5]。Wernicke脑病的预后取决于治疗的时间和维生素B_1_的补充剂量，治疗延误或维生素B_1_剂量不足，可导致不可逆的神经功能损伤，或者发展为Wernicke-Korsakoff综合征，严重者可出现昏迷、休克，甚至死亡。临床一旦确诊或疑似时，应立即给予维生素B_1_治疗，在不可逆性脑损害发生前早期识别和治疗，可有效阻止和逆转病情进展，部分患者可完全恢复正常。而对于维生素B_1_最佳的给药剂量、给药方式、给药时间及治疗持续时间，目前国内尚无统一的标准和指南推荐，也缺乏随机对照试验的证据[15]。ENFS推荐维生素B_1_ 200 mg加入到100 ml生理盐水或5％葡萄糖中静脉滴注每日3次，给药时间超过30 min。ENFS同时指出，维生素B_1_静脉滴注的整体安全性非常好，仅少数患者出现轻微的过敏反应。英国皇家内科医学院（RCP）推荐酒精性Wernicke脑病的患者采用更大剂量的维生素B_1_（500 mg每日3次静脉滴注）[16]。杨文利等[17]报道了2例HSCT后合并Wernicke脑病的儿童患者，予以维生素B_1_ 100 mg每日2次静脉滴注治疗，3 d后症状明显好转。Kuo等[18]认为，肿瘤患者在确诊或怀疑Wernicke脑病时即应给予维生素B_1_ 500 mg每日3～4次静脉滴注或肌肉注射，症状好转后继续予以250 mg/d治疗3～5 d。我们报道的3例患者，短时间内出现眼球震颤、情感淡漠及嗜睡、昏迷等症状，病情急剧进展。我们采用ENFS推荐的维生素B_1_的剂量和给药途径，3例患者均在补充维生素B_1_后短时间内明显好转，仅遗留轻度记忆力减退或轻微眼球震颤。目前尚无指南将Wernicke脑病作为HSCT后的CNS并发症单独列出，维生素B_1_的给药途径、应用剂量及疗程也无统一共识推荐，大多数都是经验性治疗。我们认为，尽早静脉大剂量补充维生素B_1_对于疗效获得和预后改善极为关键，合适的维生素B_1_剂量及疗程、以及对于移植后营养状态差的患者预防性应用维生素B_1_是否必要，尚需进一步研究。

综上，本组3例Wernicke脑病患者的诊疗经过提示，HSCT患者一旦出现神经、精神症状，应警惕Wernicke脑病的发生，应及时进行头颅MRI检查，争取做到早期诊断、早期治疗，以降低死亡率、减少神经系统后遗症。
